# Effective communications strategies to increase the impact of environmental health research

**DOI:** 10.1186/s12940-023-00997-6

**Published:** 2023-07-18

**Authors:** Rebecca E. Fuoco, Carol F. Kwiatkowski, Linda S. Birnbaum, Arlene Blum

**Affiliations:** 1 Green Science Policy Institute, Berkeley, CA USA; 2 Department of Biological Sciences, North Carolina State University, Raleigh, USA; 3 Scientist Emeritus, National Institute of Environmental Health Sciences, Research Triangle Park, NC USA; 4 Nicholas School of the Environment, Duke University, Durham, NC USA; 5 Department of Cell and Molecular Biology, University of California, Berkeley, USA

**Keywords:** Science communication, Media, Scholarly impact, Policy, Per- and polyfluoroalkyl substances (PFAS)

## Abstract

**Background:**

Per- and polyfluoroalkyl substances (PFAS) are the subject of a growing body of research with the potential to positively impact public and ecological health. However, to effect positive change, findings must be communicated beyond the scientific community.

**Objective:**

We sought to (a) evaluate the relationships between communications strategy, media attention, and scholarly citations of PFAS research and (b) offer guidance for researchers and communications professionals who would like to publicize future work and increase its impact.

**Methods:**

We analyzed 273 peer-reviewed epidemiological studies on PFAS human health impacts with publication years 2018–2020, as collected by a pre-existing database. We investigated whether a press release was issued, open-access status, abstract and press release readability, timing of publication and press release distribution, journal impact factor, study type and sample size, statistical significance of finding(s), number of scholarly citations, and the Altmetric Attention Score (a measure of media attention).

**Discussion:**

Of papers reporting a statistically significant association with health harm, those with a press release received 20 times more media attention (as assessed by Altmetric scores) than those that did not. However, only 6.2% of all papers and 7.8% of significant papers issued one. Among papers with a press release, media attention was positively correlated with better abstract and press release readability and speed in issuing the press release. Scholarly citations were positively correlated with media attention, presence of a press release, and open-access status.

**Conclusion:**

Most papers with significant findings on PFAS are published without a press release and receive little or no media attention. This reduces the likelihood that important research is reaching the public and decisionmakers who can translate science into action. Issuing a press release and receiving media attention also appear to increase scholarly citations. We provide recommendations for authors to increase the reach and impact of future papers.

## Background

Peer-reviewed journal articles are critical tools for scientist-to-scientist communication, but they are generally not read or easily understood by the public, practitioners, or policymakers [1–4]. For the environmental health field, this can represent a lost opportunity to inform government regulations, business practice, healthcare decisions, and individual and community action to protect health.

Sharing research with the news media is an effective way to close this gap and reach audiences that can translate findings into action [5]. Indeed, a diverse body of research suggests media coverage of health research influences policy [6, 7], medical practice [8–11], and consumer behavior [12–17]. Some studies have found direct correlations between media reporting and public health outcomes [18–20]. Of course, not all studies—even those with scientifically important findings—warrant a press release. Nevertheless, we see opportunities for more press releases to facilitate greater impact and translation of environmental health research.

In the authors’ own experience, media coverage of our research on harmful flame retardants in furniture and children’s products was pivotal to policy changes that led to the chemicals no longer being used in these products in North America [21–23]. Similarly, we have seen legislative movement and market changes spurred by the wide media coverage of a recent study finding per- and polyfluoroalkyl substances (PFAS) in cosmetics [24–26].

We set out to understand what factors influence the amount of media attention an environmental health paper receives, and whether the same factors influence the scholarly impact of papers. For our study sample, we focused on human epidemiology research on PFAS exposure, as it is an area of high current scientific and public interest in addition to being an area of expertise of this author group. We use our results to issue evidence-based guidance for environmental health scientists to increase the impact of their work through media outreach.

## Methods

### Article selection

We selected all peer-reviewed human studies on PFAS with publication years 2018–2020 included in the PFAS-Tox Database, a systematic evidence map of 29 PFAS available at https://pfastoxdatabase.org [27].

This database is an exhaustive collection of the PubMed-indexed literature on the physiological health effects of these compounds, individually or combined. It should be noted that the database does not include studies that only measured PFOA or PFOS, two widely studied chemicals in the large class of PFAS.

Screening for inclusion in the database was based on the following criteria: Any health outcome or type of biological response measured in human, animal (whole organism including experimental and observational studies), or ex vivo/in vitro models utilizing organs, tissues, cell lines, or cellular components (e.g. cell-free receptor binding assays). Studies that did not include a health outcome such as biomonitoring, wildlife detection, metabolic fate, or ecotoxicology were excluded. [28]. The database also excludes studies that did not contain original data, such as reviews, editorials, and commentaries. We analyzed only human studies from this database as journalists consider them more newsworthy than other study types [29]. We limited our analysis to the most recent three-year period available to reduce confounding from the rapidly changing media landscape [30].

### Study variables

For each article, the following variables were obtained: the journal impact factor, study type, sample size, whether the authors reported a statistically significant link between the studied compound(s), and at least one adverse health outcome. We hypothesized that studies with statistically significant findings were more likely to attract attention from journalists than those that reported null or non-statistically- significant results.

### Communication variables

For each article we also obtained the following variables related to communications efforts (i.e., within control of the author and/or journal after acceptance): whether or not the article was published open-access by the journal (note that we did not differentiate between open-access journals versus those who offer it as an option), the day of week it was published online, and the readability of the abstract as calculated by the Simple Measure of Gobbledygook (SMOG Index). The SMOG Index is calculated based on the total number of polysyllables and total sentences, with the score directly correlating with US grade level [31]. It is widely considered the gold standard for evaluating the readability of health information [32].

We also checked whether articles issued a press release by searching EurekAlert.org (an online free database for science press releases hosted by the American Association for the Advancement of Science), the lead institution’s website, and Google. For those that did have a press release available online, we recorded the date the press release was issued and used the SMOG Index to calculate its readability [31].

### Measuring media attention

We used Altmetric’s web application, which tracks the number of mentions an article has received in news outlets, blogs, social media (Twitter, Facebook, Reddit, etc.), and policy documents through its digital object identifier (DOI) [33]. The application derives an Altmetric Attention score from an automated algorithm and represents a weighted count of the online media attention by source [34]. For example, news articles are weighted more heavily than posts on Twitter. Higher Altmetric Attention scores indicate higher levels of media attention. Scores were recorded on March 1, 2022 (over two years after the most recent study publication date). Since the vast majority of attention occured within the first week papers were published, there was no adjustment for publication age.

### Measuring scholarly impact

We recorded Google Scholar’s “cited by” count for each paper on August 8, 2022. We then divided the count by the number of days since publication for an age-adjusted citation count.

### Analysis

We report descriptive statistics of all variables for the full set of articles. We performed ANOVA F-tests to determine the impact of categorical variables (study type, open-access status, publication day of week, whether or not a statistically significant association with an adverse health effect was reported, the presence or absence of a press release, whether there was a delay in issuing the press release) on Altmetric scores. We then calculated the Pearson correlation coefficients between the Altmetric scores and numeric variables (journal impact factor, study sample size, abstract readability, press release readability). We also performed ANOVA F-tests to determine the impact of categorical communications variables (open-access status, the presence or absence of a press release) on scholarly citations. We then calculated the Pearson correlation coefficient between the Altmetric scores and citations. Statistical significance was assessed as p < 0.05.

## Results

### Study and communication variables

As of January 5, 2022, there were 273 peer-reviewed human epidemiology articles with publication years 2018–2020 in the PFAS-Tox Database. The articles were published in 59 journals with journal impact factors ranging from 0.296 to 19.11 (Table 1). Nearly half of the study designs were cross-sectional, more than a third were cohort, and the remainder were case-control, randomized control trial, or ecological. Study sample sizes ranged from 20 to 105,114.

**Table 1:** Descriptive Statistics for Study and Communication Variables, All Studies (n = 273)

NUMERIC VARIABLE	MEAN	RANGE
Journal Impact Factor	7.1	0.3–19.1
Sample Size	2,251	20–105,114
Abstract SMOG Index Reading Grade Level	11.1	1.8–18.5
Press Release SMOG Index Reading Grade Level	11.9	9.2–14.8
Days Between Publication and Press Release	34	0–213
Scholarly Citations	24	0-116
**CATEGORICAL VARIABLE**	**NUMBER**	**PERCENTAGE**
Open-Access	127	46.5%
Study Type, n (%)		
Cross sectional	133	48.7%
Cohort	96	35.2%
Case control	36	13.2%
Randomized control trial	6	2.2%
Ecological	2	0.7%
Publication Day of Week		
Monday	50	18.3%
Tuesday	59	21.6%
Wednesday	45	16.5%
Thursday	48	17.58%
Friday	39	14.3%
Saturday	24	8.8%
Sunday	8	2.9%
Significant Finding		
Yes	205	75%
No	68	25%
Press Release		
Yes	17	6.2%
No	256	93.8%

Three-quarters of the articles reported a statistically significant link between the studied compound(s) and at least one adverse health outcome. These included respiratory, reproductive, neurological, immune, liver, kidney, and cancer outcomes.

Almost half were published open-access by the journal. Most were published on a weekday, with less than 10% published on a Saturday or Sunday. The mean abstract reading grade level (as calculated by the SMOG Index) was 11.14.

Only 17 articles (6.23%) had a press release. Even in the subset of articles reporting at least one statistically significant adverse health outcome, only 16 (7.8%) had a press release. The significant adverse health outcomes reported by the 16 papers included miscarriages, preeclampsia, lower birth weight, reduced fetal growth, earlier menopause, lower IQ, weight gain and regain, more severe COVID-19, metabolic changes in pregnancy, risk of Type 1 Diabetes, risk and accelerated development of celiac disease, cavities, and shorter telomere length. Some of these outcomes were associated with individual PFAS, while others were associated with mixtures of multiple PFAS and/or mixtures of PFAS and other pollutants. The strengths of the reported associations (as characterized by each paper’s authors) varied widely and did not appear to differ from those of articles without press releases.

The mean SMOG Index reading grade level of the press releases was 11.91, which was slightly higher than that of the abstracts of the papers themselves. About one-third of the press releases were issued on the same day the paper was published online, another third were issued one to four days later, and the remaining third were issued multiple weeks or months later.

### Impacts on media attention

The mean Altmetric Attention score of the 273 studies was 16.69, with a median of 2, and a range of 0 to 734. A quarter of the articles had a score of 0, meaning the Altmetric web application detected no news articles, blogs, or social media posts associated with the article. Another quarter had a score of 1, typically meaning there was a social media post but no news articles or blogs. Another quarter had a score between 2 and 9. The highest quartile had scores ranging from 10 to 734, with most under 100.

The variable most strongly and significantly linked to media attention was whether a press release was issued (Table 2). The mean Altmetric score of articles with press releases was 156, which was more than 20 times higher than those without press releases.

**Table 2:** Impact of Study and Communication Variables on Altmetric Score, All Studies (n = 273)

VARIABLE	MEAN ALTMETRIC SCORE	TEST STATISTIC (Pearson’s r or F-test)	P-VALUE
STUDY VARIABLES			
Journal Impact Factor	N/A	r = 0.16	0.01
Sample Size	N/A	r = -0.04	< 0.001
Study Type		F = 7.43	< 0.001
Cross sectional	14.9		
Cohort	13		
Case control	11.9		
Randomized control trial	144.8		
Ecological	14		
Significant Finding		F = 4.06	0.045
Yes	21		
No	3.8		
**COMMUNICATION VARIABLES**			
Open Access		F = 3.25	0.07
Yes	23.8		
No	10.5		
Publication Day of Week		F = 0.32	0.93
Monday	20.6		
Tuesday	20.5		
Wednesday	12.8		
Thursday	21.7		
Friday	9.9		
Saturday	11.9		
Sunday	3.9		
Abstract SMOG Index Reading Grade Level	N/A	r = 0.08	0.13
Press Release		F = 144.14	< 0.001
Yes	156		
No	7.4		

Another important factor was whether the article reported at least one statistically significant association with an adverse health effect. The mean Altmetric score of those that did was nearly six times higher than those that did not.

Open-access papers received twice as much media attention as those that were paywalled. However, the relationship was of borderline statistical significance. Another communication factor that appeared to boost media attention was publishing on a Tuesday, followed by Thursday and Monday. The mean media attention score for articles published on these days was nearly double that of papers published on Friday or during the weekend. However, the relationship was not statistically significant. Abstract readability did not appear to have an effect.

Study type and journal impact factor also influenced media attention. The mean Altmetric score of randomized control trials was about 10 times higher than other study designs (note however that only 2% of studies were randomized control trials, and this small sample size may lead to unreliable effects estimates). Journal impact factor was weakly but significantly positively correlated with media attention.

Because there was likely some degree of self-selection of more inherently newsworthy papers, we did a separate analysis of the subset of 16 papers that had a statistically significant finding and issued a press release (Table 3) in order to identify which strategies made an impact in conjunction with a press release. Study type did not have a statistically significant impact, but journal impact factor, abstract readability, press release readability, and speed in issuing a press release did. There was a strong negative relationship between the abstract SMOG Index reading grade level (e.g., better abstract readability was linked to more media attention). The readability of the abstract was more important than the readability of the press release, which had a weaker but significant correlation with media attention. It’s notable that abstract readability was important for papers with press releases but not for the whole set of papers.

**Table 3:** Impact of Study and Communication Variables on Altmetric Score, Significant Studies with Press Release (n = 16)

VARIABLE	MEAN ALTMETRIC SCORE	TEST STATISTIC (Pearson’s r or F-test)	P-VALUE
STUDY VARIABLES			
Journal Impact Factor	N/A	r = 0.3	0.006
Sample Size	N/A	r = -0.06	0.002
Study Type		F = 2.16	0.139
Cross sectional	225.8		
Cohort	90.6		
Case control	34.5		
Randomized control trial	406.5		
**COMMUNICATION VARIABLES**			
Open Access		F = 0.16	0.7
Yes	178.8		
No	135.6		
Publication Day of Week		F = 0.70	0.63
Monday	174.5		
Tuesday	384		
Wednesday	91.3		
Thursday	186.2		
Friday	52.5		
Saturday	55		
Abstract SMOG Index Reading Grade Level	N/A	r = -0.48	0.007
Press Release SMOG Index Reading Grade Level	N/A	r = -0.18	0.007
Days Between Publication and Press Release (if issued)		F = 6.34	0.01
Same Day	235.2		
One Day	149.3		
>One Day	36.7		

Longer delays between publication and press release distribution were negatively correlated with media attention.

### Impacts on Scholarly Citations

The mean total citation count at the time of analysis was 24 articles. Issuing a press release and publishing open-access were both significantly and positively associated with citation counts adjusted for the number of days since publication (Table 4). The mean age-adjusted citation count for papers with press releases was two-thirds higher than those without. There was also a significant positive correlation between citations and Altmetric scores.

**Table 4:** Impact of Communication Variables on Scholarly Citations, All Studies (n = 273)

VARIABLE	MEAN AGE-ADJUSTED CITATION COUNT (Citations/Day)	TEST STATISTIC (Pearson’s r or F-test)	P-VALUE
Open Access		F = 10.15	0.002
Yes	0.024		
No	0.019		
Press Release		F = 15.29	< 0.001
Yes	0.034		
No	0.020		
Altmetric Score	N/A	r = 0.4	< 0.001

## Discussion

By far the most important factor determining media attention in our analysis was issuing a press release. Our results are consistent with Haneef et al.’s analysis of cancer treatment studies, which found that the presence of a press release was the most significant determinant of media attention [35]. Other communication variables that appeared to positively influence media attention were publishing Monday, Tuesday, or Thursday and publishing open-access.

For articles with press releases, better abstract readability was strongly and positively associated with media attention, appearing to be more important than the readability of the press release itself. Issuing a press release swiftly after publication—ideally on the same day—was also an important determinant of media attention.

Issuing a press release, publishing open-access, and gaining media attention were also positively and significantly associated with more scholarly citations. This suggests that these communication efforts increase the reach among both scientific and non-scientific audiences.

### Limitations

One limitation of our analysis was that the more inherently newsworthy papers may have self-selected in issuing press releases. Quantitatively or systematically evaluating the news and scientific value of the papers was not feasible given that the numerous variables at play are often subjective. However, we did identify a number of articles that did not issue press releases that, by our judgement, were of similar or higher news or scientific value (in terms of study sample size, strength and significance of reported correlations, and severity or level of public concern related to the outcomes) than those that did. For example, articles without press releases included studies reporting “a nearly 2-fold increase in risks of preterm birth [36],” “increased odds of ovarian and breast cancers with a positive dose-response relationship [37],” a “2.3-96-fold increase in odds of diagnosis for osteoporosis [38],” “a substantially higher [gestational diabetes] risk [39],” and “a relationship between low-dose background PFAS exposure and altered liver function in the general population [40].” All five of these articles had Altmetric Attention scores between 0 and 7. This is consistent with research finding that Altmetric Attention scores are not necessarily reflective of study quality [41]. One cause of the discrepancy may be that outcomes of such studies already have substantial medical literature, making the studies less novel to the scientific community. However, a lack of novelty to the scientific community does not equate to a lack of novelty to the general public and decisionmakers. Any strong study showing a significant link to harm—even if that harm is already recognized by researchers in the field—is a communication opportunity to bridge the gaps between science, policy, and practice. Notably, authors should use good judgment as to whether to promote certain studies, such as those with conflicting evidence, designs that are inadequate to infer causation, or endpoints with plausible reverse causation, to lay audiences who do not have the scientific expertise to interpret the findings appropriately.

Another limitation is that we are unable to measure the extent of control that authors have on the readability (as measured by the SMOG Index) of individual papers. Some areas of research are inherently more technical and less easily converted to lay language than others.

Other limitations include that our sample of articles issuing press releases was small, we did not have information about whether articles or press releases were shared with reporters under embargo, we could not account for press releases that may have been distributed via email but are not available online, and the Altmetric web application may miss media mentions that do not include a link to the article.

We should also note that our analysis—and therefore recommendations—may be biased toward U.S. and other English-language media. Altmetric does count non-English media articles, but is unclear how representative it is. Further, most journal articles and press releases are issued in English, which may limit coverage in non-English media outlets.

### Barriers to improved research communications

A common barrier to research communications is a fear among scientists that press coverage may be inaccurate or over-hyped. This is an important concern, however, Sumner et al. found that overstatements can often be traced back to university press releases [42]. This suggests that the solution is for scientists to take a more active role in press release drafting and carefully ensure their accuracy rather than forego issuing one at all. Multiple studies have found that more-accurate press releases don’t receive less coverage, and that the coverage is more correct [43, 44]. The onus for better scientist participation in and accuracy of press releases is shared by the scientists themselves and their press officers and institutions.

Another barrier is often a real or perceived lack of career incentive to focus on non-scholarly communications. However, our analysis found that communications and media attention increase an article’s citations in the scientific literature. Further, there is growing recognition that the reduction of scientific achievement to traditional metrics like number of scientific journal publications may have harmful effects on research and its practitioners [45]. It’s also important to note the balance of incentives varies by type, size, and country of author institutions, which we did not analyze in this study due to the high frequency of mixed author groups.

.

Other barriers include a lack of time on the part of the research team and/or institutional press offices, a lack of funding to cover open-access fees, a lack of media savvy among the research team, and differing philosophical views about the role of scientists in society. We plan to conduct research into these and other barriers and how to overcome them.

We also recognize that effecting change is not the only motivation for scientific endeavors. Intellectual curiosity and advancing science for its own sake are common reasons for conducting research. However, further understanding and improving the connection between science and media coverage has benefits in both directions. For example, more attention to PFAS research can increase funding for more scientific studies, create new opportunities, and attract new talent to the field.

### Recommendations

While not all journal articles merit a press release, we find that important information in many newsworthy and potentially impactful environmental health studies is not leaving the confines of peer-reviewed journals. The scientific and news values of a study are subjective and distinct determinations, and we encourage scientists to consult their institution’s press office or expert colleagues for help making those judgment calls.

Based on our analysis and experience, we issue the following five key recommendations for a press strategy to increase the reach and impact of journal articles:

1. Have a press strategy and issue a press release following the guidance of an experienced communications officer [46].
2. Share the press release with trusted reporters under embargo a few days to a week *before* the article goes online [47]. This can be done via email and/or through services like EurekAlert! [48].
3. Ask the journal to set the online publication date between Monday and Thursday.
4. Ensure your abstract clearly communicates the significance and implications of your research and is as readable as possible. You can find free web-based readability calculators online [49].
5. Publish open-access. Advances in article processing charge funding mechanisms are making this more attainable for some authors, although pervasive inequities remain [50].To address this disparity, more funding agencies should offering to help cover open-access fees and more journals should offer lower-cost or free open-access publishing.

## Data Availability

Data from the 273 studies analyzed was collected from the PFAS-Tox Database, available at https://pfastoxdatabase.org. The amended dataset with Altmetric scores, scholarly citations, press release information, and readability scores are available from the corresponding author on reasonable request.

## Abbreviations

PFAS: Per- and polyfluoroalkyl substances
SMOG: Simple Measure of Gobbledygook
